# Integration of optic flow into the sky compass network in the brain of the desert locust

**DOI:** 10.3389/fncir.2023.1111310

**Published:** 2023-04-28

**Authors:** Frederick Zittrell, Kathrin Pabst, Elena Carlomagno, Ronny Rosner, Uta Pegel, Dominik M. Endres, Uwe Homberg

**Affiliations:** ^1^Department of Biology, Philipps-Universität Marburg, Marburg, Germany; ^2^Center for Mind, Brain and Behavior (CMBB), University of Marburg and Justus Liebig University, Marburg, Germany; ^3^Department of Psychology, Philipps-Universität Marburg, Marburg, Germany

**Keywords:** optic flow, sky compass, desert locust, orientation, computational model, central complex, head direction, intracellular recordings

## Abstract

Flexible orientation through any environment requires a sense of current relative heading that is updated based on self-motion. Global external cues originating from the sky or the earth‘s magnetic field and local cues provide a reference frame for the sense of direction. Locally, optic flow may inform about turning maneuvers, travel speed and covered distance. The central complex in the insect brain is associated with orientation behavior and largely acts as a navigation center. Visual information from global celestial cues and local landmarks are integrated in the central complex to form an internal representation of current heading. However, it is less clear how optic flow is integrated into the central-complex network. We recorded intracellularly from neurons in the locust central complex while presenting lateral grating patterns that simulated translational and rotational motion to identify these sites of integration. Certain types of central-complex neurons were sensitive to optic-flow stimulation independent of the type and direction of simulated motion. Columnar neurons innervating the noduli, paired central-complex substructures, were tuned to the direction of simulated horizontal turns. Modeling the connectivity of these neurons with a system of proposed compass neurons can account for rotation-direction specific shifts in the activity profile in the central complex corresponding to turn direction. Our model is similar but not identical to the mechanisms proposed for angular velocity integration in the navigation compass of the fly *Drosophila*.

## 1. Introduction

Animals navigate to feed, escape, migrate, and reproduce. Navigational tasks require a sense of current travel direction, which must be anchored to external cues and updated by internal cues, generated by ego-motion. Celestial cues are used as external cues by many insects, such as bees (von Frisch, 1946), ants (Fent, 1986), butterflies (Perez et al., 1997), dung beetles (Byrne et al., 2003), fruit flies (Weir and Dickinson, 2012), and certain lepidopteran caterpillars (Uemura et al., 2021). The sun and the skylight polarization pattern provide a reliable reference for dead reckoning (Gould, 1998). Internal cues that monitor self-motion, such as proprioceptive feedback (Wittlinger et al., 2006), and optic flow (Srinivasan, 2015; Stone et al., 2017) provide information about traveling speed and covered distance and may update the inner sense of direction in the absence of external cues. Only the flexible combination of information from external and internal cues enables robust and efficient navigation behavior, such as path integration (Heinze et al., 2018).

The central complex (CX), a midline spanning group of neuropils, houses the sense of direction in the brain of insects. It consists of the protocerebral bridge (PB), the lower (CBL) and upper (CBU) division of the central body, also termed ellipsoid body (EB) and fan-shaped body (FB), and a pair of layered noduli (NO), and is associated with behavioral decisions related to spatial orientation (Pfeiffer and Homberg, 2014). The PB and the CBL are subdivided into series of 16 or 18 columns that are connected across the brain midline in a precise topographic manner (Pfeiffer and Homberg, 2014; Hulse et al., 2021; Homberg et al., 2022).

CX neurons in various insect species are tuned to celestial cues (Heinze and Homberg, 2007; Heinze, 2017; Honkanen et al., 2019). Evidence from the fly *Drosophila* (Hardcastle et al., 2021) and the desert locust (Pegel et al., 2019; Zittrell et al., 2020) suggest that solar azimuth is encoded in the CX in a compass-like manner. Silencing compass neurons in the CX impairs menotactic navigation behavior in the fruit fly (Giraldo et al., 2018), showing the necessity of the CX for this behavior. Like mammalian head direction cells (Taube, 1998, 2007), specific CX neuron populations are tuned to the animal's current heading (Seelig and Jayaraman, 2015; Hulse and Jayaraman, 2020). This internal heading estimate is multimodally tethered to environmental cues, such as visual compass cues and wind direction (Okubo et al., 2020), but also operates without external input, because internal cues from self motion are likewise integrated (Green et al., 2017; Turner-Evans et al., 2017; Green and Maimon, 2018).

**Figure 1:**
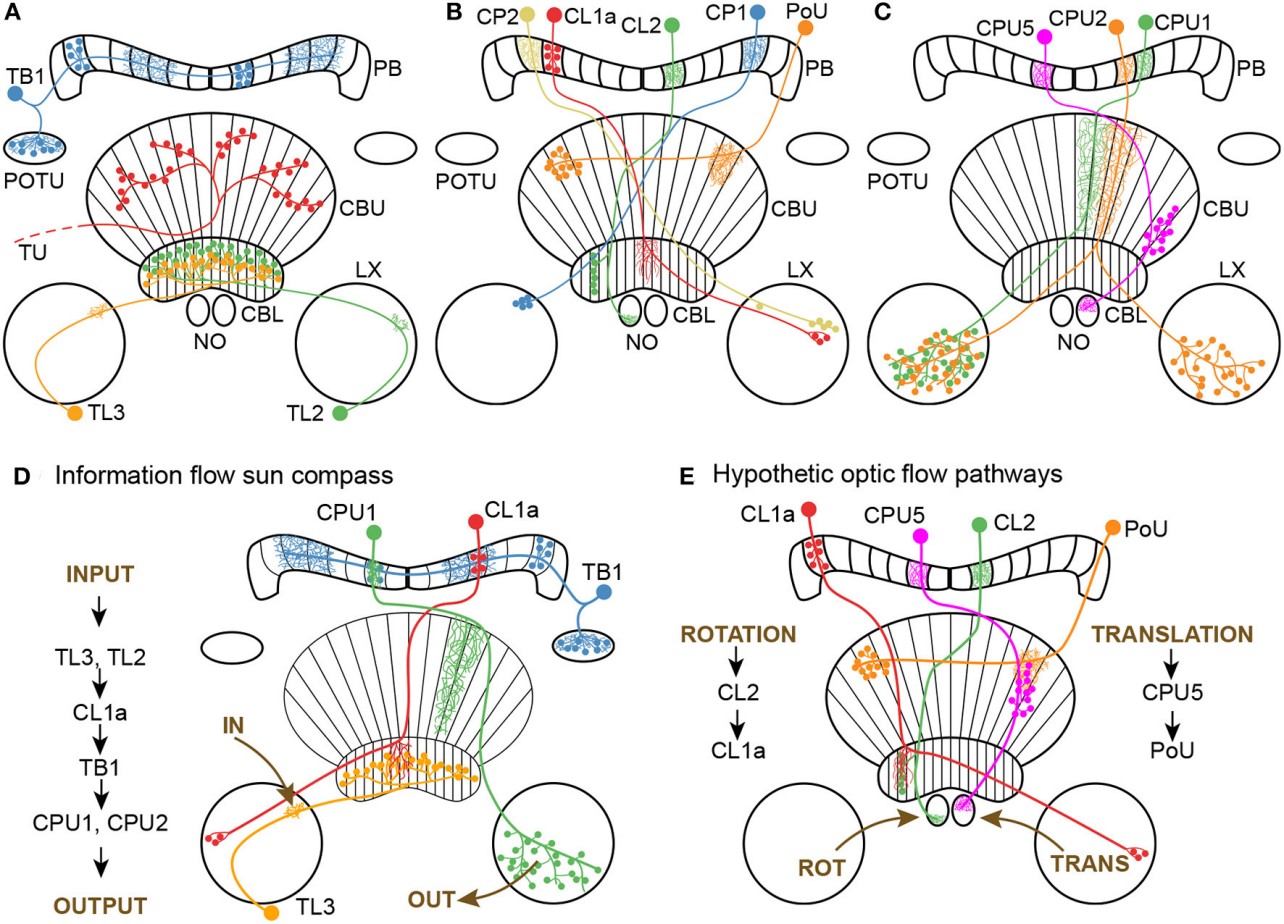
Morphology of neuron classes analyzed in this study. **(A–C)** Schematics of the locust central complex and associated neuropils (CBL, lower division of the central body; CBU, upper division of the central body; LX, lateral complex; NO, noduli; PB, protocerebral bridge; POTU, posterior optic tubercle) with individual neurons from different classes superimposed. Large dots indicate somata, small dots indicate axonal (presynaptic) arborizations, and fine lines indicate dendritic (postsynaptic) arborizations. **(A)** Tangential neurons. We classified TU neurons as a group of diverse neurons that only have in common that they have large presynaptic arborizations in the CBU and input regions outside the central complex. Wiring schematics based on von Hadeln et al. (2020). **(B, C)** Columnar neurons. Wiring schematics based on Heinze and Homberg (2008). **(D)** Information flow through core neuronal elements of the sun compass circuit in the locust central complex, based on Heinze and Homberg (2007) and Heinze et al. (2009). For reasons of simplicity TL2 and CPU2 neurons are not included in the diagram. **(E)** Hypothetic neuronal cell types shown in A-C and their putative connectivity, that might be involved in optic flow signaling. Data are based on corresponding cell types in the fly *Drosophila* (Green et al., 2017; Turner-Evans et al., 2017; Lu et al., 2022) and the sweat bee *Megalopta genalis* (Stone et al., 2017).

The cellular understanding of the CX navigation network has made considerable progress, largely owing to research in the fruit fly (Seelig and Jayaraman, 2015; Turner-Evans et al., 2017; Okubo et al., 2020; Hulse et al., 2021; Lu et al., 2022; Lyu et al., 2022), desert locust (Homberg et al., 2011, 2022), dung beetles (Dacke and el Jundi, 2018; el Jundi et al., 2019), monarch butterflies (Heinze and Reppert, 2011; Nguyen et al., 2021), and bees (Stone et al., 2017; Sayre et al., 2021). Based on these data, plausible models explaining network computations for navigation have been proposed (Stone et al., 2017; Le Moël et al., 2019; Sun et al., 2020, 2021). In the desert locust (*Schistocerca gregaria*), a long range migratory insect, sky compass signals enter the CX through tangential neurons targeting the CBL, termed TL2 and TL3 neurons (Figures 1A, D) that correspond to certain ER neurons in the fly (Homberg et al., 2022). Their postsynaptic partners, CL1a columnar neurons (E-PG neurons in the fly), connect the CBL to single columns in the PB (Figures 1B, E) and establish a 360° representation of space related to solar azimuth in the PB. Tangential neurons, termed TB1 and TB2 in the locust and Δ7 in the fly, distribute the compass signal across the columns of the PB (Figures 1A, D). They provide input to columnar CPU1 and CPU2 neurons (PFL neurons in flies) connecting single columns of the PB to wide areas in the lateral accessory lobes (Figures 1C, D), where navigation-related signals are conveyed to descending channels (Rayshubskiy et al., 2020; Homberg et al., 2022). Compass representations in the CX of the fly and the locust differ in several aspects. In *Drosophila* calcium imaging of E-PG neurons showed a flexible representation of 360° of space in the EB leading to a twofold representation of 360° across the PB (Seelig and Jayaraman, 2015; Hardcastle et al., 2021). In contrast in locusts, single-cell intracellular recordings from various types of PB neurons suggest a single 360° representation of space across the PB which is assumed to be fixed across the locust population (Heinze and Homberg, 2007; Zittrell et al., 2020). Whether these differences are related to differences in circuit architecture such as the EB in the fly being a closed toroidal structure, and the locust CBL, an open kidney-like neuropil (Pisokas et al., 2020), or differences in the analyzed cell types representing the compass remains to be seen. Research in flies and bees suggests that optic flow input is integrated in the sky compass network through the PB and/or NO (Green et al., 2017; Stone et al., 2017; Turner-Evans et al., 2017; Lu et al., 2022). In bees, inputs to the NO, termed TN neurons, provide optic-flow based speed information allowing for computation of path integration in the CX (Stone et al., 2017). In *Drosophila*, columnar neurons receiving input via the NO (from TN-type neurons) and the PB (via SpsP neurons) signal translational velocity and, by convergence on internal hΔB neurons of the FB, lead to a representation of translational velocity in world-centric space (Lu et al., 2022; Lyu et al., 2022). Whereas circuits involved in translational velocity coding involve the upper units of the NO and the CBU/FB, Green et al. (2017) and Turner-Evans et al. (2017) showed in flies, that columnar neurons innervating the lower units of the NO (P-EN neurons) are involved in angular velocity signaling and, through interaction with E-PG neurons, are suited to shift compass activity in the PB corresponding with turns of the fly during walking. Although neurons apparently homologous to optic-flow encoding neurons in bees and flies are known morphologically in locusts, such as TB7 (SpsP in flies), TN-type neurons, pontine PoU neurons (hΔ in flies), CL2 columnar neurons (P-EN in flies), CPU4 and 5 neurons (PFNd in flies; Figure 1E; Heinze and Homberg 2008; von Hadeln et al. 2020), only a single study has so far addressed the sensitivity of CX neurons of the locust to translational optic flow (Rosner et al., 2019). That study showed that neurons at all levels of the sky compass network were sensitive to translational forward motion, but often responses could not be separated from the effects of concurrently occurring leg movements that were elicited by the optic flow stimulus.

To investigate optic flow sensitivity in the CX of the locust more systematically, we recorded intracellularly from various types of CX neurons while stimulating laterally with wide-field gratings that simulated self-motion to the animal. We analyzed general motion sensitivity for translational and rotational motion directions and tested whether the neural responses to opposing motion directions were discriminated (direction selectivity).

We implemented an algorithmic model (in the sense of Marr and Poggio, 1979) of the CX circuit which integrates visual self-motion cues with head direction representation. Modeling was guided by data on two types of columnar neurons with one being sensitive to the direction of simulated horizontal turns.

## 2. Methods

### 2.1. Animals and preparation

Desert locusts (*Schistocerca gregaria*) were kept and dissected as described previously (Zittrell et al., 2020). Animals were reared in large groups (gregarious state) at 28 °C with a 12 h / 12 h light / dark cycle; adult locusts from either sex were used for experiments. Limbs and wings were cut off, the animals were fixed on a metal holder with dental wax, and the head capsule was opened frontally, providing access to the brain. The esophagus was cut inside the head, close to the mandibles, and the abdomen's end was cut off to take out the whole gut through this opening. The brain was freed of fat, trachea and muscle tissue and was stabilized with a small metal platform that was fixed to the head capsule. A chlorinated silver wire, inserted into the hemolymph surrounding the brain, served as the indifferent electrode. Shortly before recording, the brain sheath was removed at the target site with forceps, permitting penetration with sharp glass electrodes. The brain was kept moist with locust saline (Clements and May, 1974) throughout the experiment.

All animal procedures were performed according to the guidelines of the European Union (Directive 2010/63/EU) and the German Animal Welfare Act.

### 2.2. Intracellular recording and histology

Sharp microelectrodes were drawn with a Flaming/Brown filament puller (P-97; Sutter Instrument), their tips filled with Neurobiotin tracer (Vector Laboratories; 4 % in 1 mol · l^−1^ KCl) and their shanks filled with 1 mol · l^−1^ KCl. Intracellular recordings were amplified with a custom-built amplifier and digitized with a 1401plus (Cambridge Electronic Device, CED) analog-digital converter (ADC) or amplified with a BA-01X (npi electronic GmbH) and digitized with a Micro mkII with an ADC12 expansion unit (CED). Signals were monitored with a custom-built audio monitor and recorded with Spike2 (CED). Neurons were traced by electrically injecting Neurobiotin (~1 nA positive current for several minutes). Each neuron presented in this study originates from a different specimen. Brains were dissected and immersed in fixative (4 % paraformaldehyde, 0.25 % glutaraldehyde and 0.2 % saturated picric acid, diluted in 0.1 mol · l^−1^ phosphate buffered saline [PBS]) over night, followed by optional storage at 4 °C in sodium phosphate buffer until further processing. Brains were rinsed in PBS (4 × 15 min) and incubated with Cy3-conjugated streptavidin (Dianova; 1:1,000 in PBS with 0.3 % Triton X-100 [PBT]) for 3 d at 4 °C. After rinsing in PBT (2 × 30 min) and PBS (3 × 30 min), they were dehydrated in an ascending ethanol series (30 %, 50 %, 70 %, 90 %, 95 %, and 2 × 100 %, 15 min each) and cleared in a 1:1 solution of ethanol (100 %) and methyl salicylate for 20 min and in pure methyl salicylate for 35 min, to finally mount them in Permount (Fisher Scientific) between two coverslips. For anatomical analysis, brains were scanned with a confocal laser-scanning microscope (Leica TCS SP5; Leica Microsystems). Cy3 fluorescence was elicited with a diode pumped solid-state laser at 561 nm wavelength. The resulting image stacks were processed with Amira 6.5 (ThermoFisher Scientific, Waltham, MA) and Affinity Photo (Serif, Nottingham, UK). The chirality of some neurons could not be determined because multiple neurons of the same neuron class but on both brain sides were stained in these cases.

### 2.3. Experimental design

**Figure 2:**
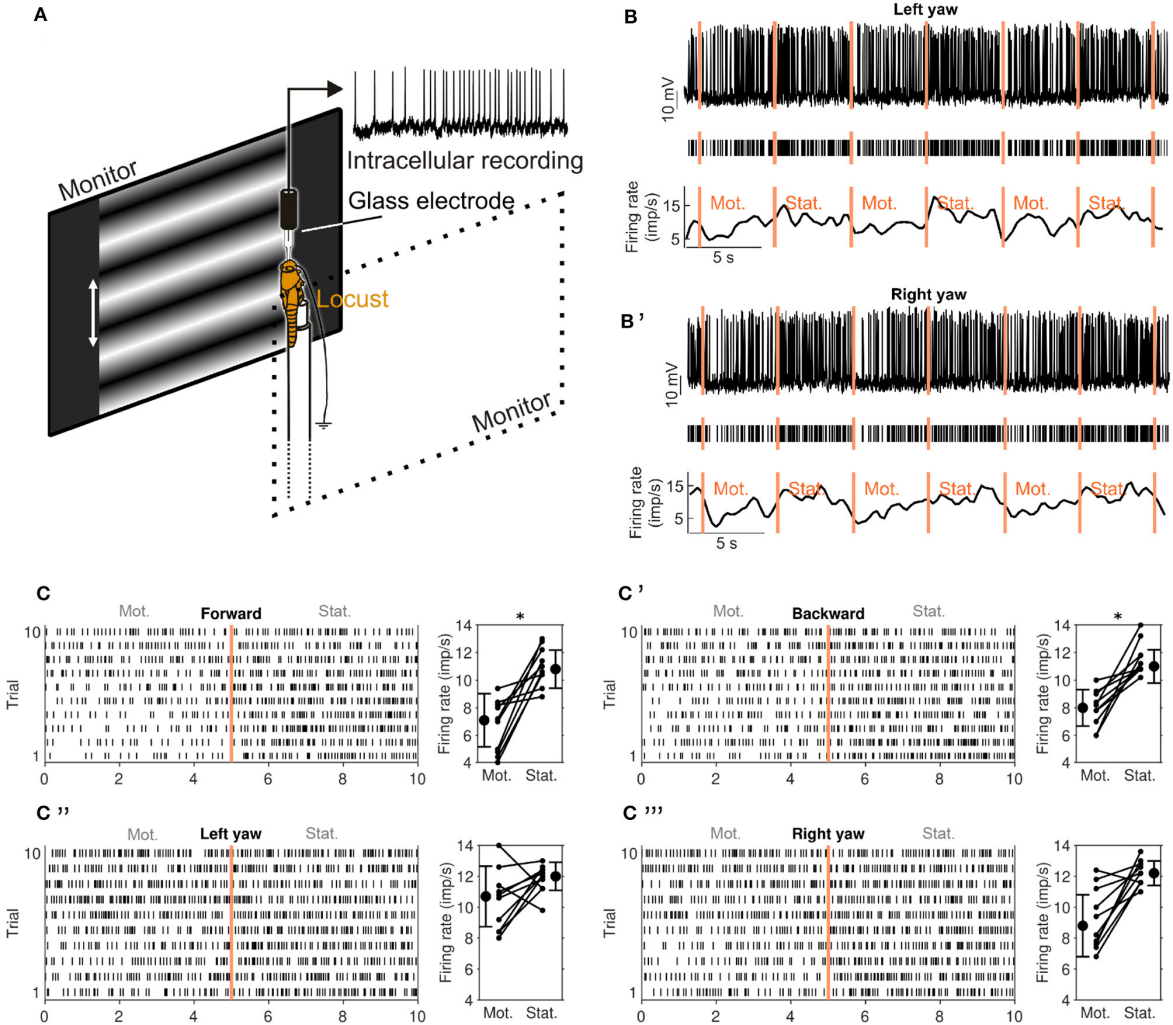
Experimental setup and visual-motion response of a CL1a neuron (neuron 550^*L*^ in Supplementary Figures S1, S2). **(A)** Animals were mounted vertically and stimulated with motion of sinusoidal grating patterns on two laterally placed monitors. **(B)** Response of a CL1a neuron to wide-field visual motion that simulated horizontal left turning (left yaw). Raw data (top), detected spikes (middle) and smoothed firing rate estimate (bottom). Vertical lines indicate onset of stimulation phases: Motion (Mot.) and stationary phase (Stat.) were alternated, each pair constituting one stimulation trial. **(B')** Same as B but for simulation of horizontal right turn motion (right yaw). **(C)** Raster plot (left) of all forward motion trials. Vertical line at 5 s indicates onset of stationary phase. Diagram on the right shows differences in firing rate between the motion (Mot) and stationary phase (Stat.) for each trial and mean firing rates for all trials. Error bars denote standard deviation. **(C'–C”')** Same as C but for **(C')** backward motion, **(C”)** left yaw and **(C”')** right yaw rotation. An asterisk indicates ‘strong evidence' in favor of the hypothesis that the firing rates differ between the motion and stationary phases (i.e., it indicates a Bayes factor ≥10 according to the conventions established by Kass and Raftery, 1995).

We used two monitors (FT10TMB, 10“, 1024x768 px at 60 Hz, Faytech, Shenzhen, China) that were placed 12.7 cm apart on the left and right side of the animal. They were mounted vertically to present sinusoidal grayscale grating patterns (Figure 2A). The displays were covered with diffuser sheets to eliminate light polarization inherent to LCD monitors. The patterns were drawn on the inner center-square (15.35 cm edge length) of the displays, covering 62.3° of the visual field on each side. The monitor brightness amounted to 1.12 · 10^11^ photons cm^−2^ · s^−1^ when displaying a black area and 7.09 · 10^13^ cm^−2^ · s^−1^ when displaying a white area. Monitor brightness was measured using a digital spectrometer (USB2000; Ocean Optics) placed at the position of the locust head.

The grating patterns were animated to simulate self-motion to the animal. We tested translational (forward and backward) motion, yaw rotation (left and right turning), lift (upward and downward), and roll (counter clockwise and clockwise). Throughout this study, these direction labels refer to simulated self-motion directions and not absolute motion of the displayed patterns. Thus, “forward motion” means that both monitors displayed a grating pattern with horizontal bands (perpendicular to the locust's body axis, cf. Figure 2A) that continuously moved from top to bottom.

Each motion direction was tested in a series of trials in pre-defined order, starting with translational motion and yaw rotation followed by lift and roll; each trial consisted of two phases, a motion phase and an immediately following stationary phase (Figures 2B, B'). All phases in the same recording lasted for 5 or 6 s. Each series consisted of two to five trials; each trial was immediately followed by the next one, unless it was the last of the series. Neurons typically responded strongly to the pattern display switch between series. Therefore, each series of a given motion direction was preceded by an adaptation phase of 5–6 s which was discarded; this phase was a single stationary phase of the same pattern used during the upcoming series, immediately followed by the first motion phase of the series. If the same motion direction was tested in more than one series, all trials were treated as if they belonged to the same series. Not all neurons could be tested for all motion directions due to recording instability.

A separate PC running MATLAB (R2019, MathWorks) with the Psychophysics toolbox (Brainard, 1997) was used to generate the grating patterns (Figure 2A). The sine gratings had a spatial resolution of 0.005 cycles · px^−1^ (one sine cycle spanned 200 px) and were shifted with 2 cycles · s^−1^ during the motion phases corresponding to a velocity of 32.5° s^−1^ in the center of the screen. These parameters are well within the range of motion stimuli eliciting optomotor responses in tethered flying locusts (Thorson, 1964; Preiss and Spork, 1995). The PC was USB-connected to an Arduino Uno (Arduino) via which TTL pulses were sent to the ADC, recorded at 500 Hz. These pulses indicated grating pattern animation and onset of stimulation phases. Two squares with 30 px edge length in the top left corner of each display were used to indicate the presented motion type by flashing them white: Each motion type was assigned a distinct number of flashes (20 ms duration) that were generated at the end of the adaptation phase of each series. Each square was covered by a photo diode that picked up the white flashes and whose signal was recorded by the ADC at 200 Hz. This allowed for encoding the motion type of each stimulation series in the data file. The generation of each rectangle flash was also recorded via the Arduino as a TTL rectangle pulse of the same duration, which allowed for measuring the precise timing of stimulus display by cross correlating diode signal and TTL signal.

### 2.4. Statistical analysis

Spikes were detected by median filtering (500 ms window width) the voltage signal and applying a manually chosen threshold. Spikes and non-spikes (gaps) within 2 ms time bins were counted during the whole 5 s long interval of each trial of stimulation condition. We chose 2 ms time bins for this analysis because this is the approximate length of the refractory period of the neurons.

In the following, we describe our design of a Bayesian analysis of motion sensitivity and direction selectivity. This analysis allows us to compute statistics on the quantities of interest directly, rather than testing against a distributional assumption that has no clear relationship to the data generating process, such as a t-statistic. Furthermore, the Bayesian approach guarantees internal consistency when multiple statistics on the same data are computed. These epistemic advantages are empirically backed by the observation that standard *t*-test statistics yielded very noisy and correspondingly uninterpretable results on our data. Lastly, Bayesian approaches will yield results on small samples, albeit at the cost of increased uncertainty in the conclusions. All computations were performed with the Python programming language (version 3.10.8) and the PyTorch (version 1.13.0) and Pandas (version 1.5.2) libraries. Plots were created with the Matplotlib library (version 3.6.2).

#### 2.4.1. Motion sensitivity

We define motion sensitivity as a neuron's property to have different firing rates during motion and stationary phases. We analyzed motion sensitivity for each tested neuron and motion direction by comparing the neuron's firing rate during the motion phase with that during the following stationary phase. Firing probabilities were computed by integrating prior knowledge about compass neuron activity in general and the condition-specific data from each neuron via Bayesian inference. For each neuron *n*, we computed a posterior over three different hypotheses: First, that the firing probability in 2 ms time bins during the motion phase *r*_*m*_ is lower than the firing probability *r*_*s*_ during the stationary phase, *H*(*r*_*m*_ < *r*_*s*_), second, that the firing probabilities are equal *H*(*r*_*m*_ == *r*_*s*_), or third, that *r*_*m*_ exceeds *r*_*s*_, *H*(*r*_*m*_ > *r*_*s*_). A high posterior for the first or third hypothesis would indicate motion sensitivity, while a high posterior for the second hypothesis would indicate that the neuron does not respond to the motion stimulation.

Using Bayes' rule, we computed the posterior distribution *P*(*H*|*D*) over the three hypotheses *H* ∈ {*H*(*r*_*m*_ < *r*_*s*_), *H*(*r*_*m*_ == *r*_*s*_), *H*(*r*_*m*_ > *r*_*s*_)} given the experimental data *D*, assuming a uniform hypothesis prior, a Bernoulli observation model and a joint Beta prior for the firing probabilities. This joint prior was restricted by the firing probability constraints expressed in each hypothesis, e.g. for *H*(*r*_*m*_ < *r*_*s*_), the probability *P*(*r*_*m*_ ≥ *r*_*s*_) = 0 etc. For details, see Supplementary material section ‘Statistical Model and Power Analysis of Motion Sensitivity'.

To summarize the information embedded in this posterior and to simplify comparison across multiple neurons, we computed two scores: First, the Bayes factor *BF*_≠_ in favor of *r*_*m*_ ≠ *r*_*s*_:


(1) $$\begin{array}{l} BF_{\neq }=\frac{P\left(H\left(r_{m}<r_{s}\right)|D\right)+P\left(H\left(r_{m}>r_{s}\right)|D\right)}{P\left(H\left(r_{m}==r_{s}\right)|D\right)}. \end{array}$$

We plotted an asterisk in Figures 2, **4** whenever *BF*_≠_ ≥ 10 which indicates 'strong evidence' in favor of unequal firing rates (Kass and Raftery, 1995). Second, we evaluated a single motion sensitivity score (MSS) per neuron and motion direction (dir):


(2) $$\begin{array}{l} MSS_{dir}=\{\begin{array}{cc} H\left(r_{m}>r_{s}\right) & :\;1\\ H\left(r_{m}==r_{s}\right) & :\;0\\ H\left(r_{m}<r_{s}\right) & :\;-1 \end{array} \end{array}$$

We weight this score with the corresponding hypothesis posterior probability and sum across all neurons of one type. The maximal value for one firing probability hypothesis is therefore equal to the number of neurons of a given type.

Further, we computed absolute motion sensitivity scores (AMSS) for four motion categories (cat), each comprised of two opposing motion directions *A* and *B*: translational motion (forward or backward direction), yaw rotation (left or right turning), lift (upward or downward), and roll (counterclockwise or clockwise):


(3) $$\begin{array}{l} AMSS_{cat}=1-\left[P\left(H\left(r_{m,A}==r_{s,A}\right)|D\right)*P\left(H\left(r_{m,B}==r_{s,B}\right)|D\right)\right] \end{array}$$

where *r*_*m,A*_ and *r*_*m,B*_ are firing probabilities during stimulation with opposing motion directions in the respective motion category. In other words, this score will be close to one if at least one motion direction of a category elicits a strong deviation from the stationary firing probability. We sum this score across all neurons of a given type.

#### 2.4.2. Direction selectivity

We define direction selectivity as a neuron's property to respond contrarily to two opposing motion directions *A* and *B*. We analyzed direction selectivity in the four motion categories outlined above: translation, yaw rotation, lift, and roll. In the following, the hypothesis *H*(*r*_*m,A*_ ≥ *r*_*s,A*_) = *H*(*r*_*m,A*_ > *r*_*s,A*_) ∨ *H*(*r*_*m,A*_ == *r*_*s,A*_) where ∨ indicates a logical 'or', and ∧ is a logical 'and'.

We compute a direction selectivity score as


(4) $$\begin{array}{l} DSS_{cat}=\{\begin{array}{l} \left[H\left(r_{m,A}\geq r_{s,A}\right)\wedge H\left(r_{m,B}<r_{s,B}\right)\right]\vee [H\left(r_{m,A}>r_{s,A}\right)\\ \;\wedge H\left(r_{m,B}==r_{s,B}\right)]\;:\;1\\ \left[H\left(r_{m,A}<r_{s,A}\right)\wedge H\left(r_{m,B}\geq r_{s,B}\right)\right]\vee [H\left(r_{m,A}==r_{s,A}\right)\\ \;\wedge H\left(r_{m,B}>r_{s,B}\right)]\;:\;-1\\ \text{ otherwise }:\;0 \end{array} \end{array}$$

For example, *DSS*_*translation*_ is +1(-1) if the firing probability does not decrease during forward (backward) motion and decreases during backward (forward) motion, or if it increases during forward (backward) motion and does not change during backward (forward) motion. It is 0 if the firing probability changes in the same direction for both motion directions. We weight this score with the corresponding hypothesis posterior probability and sum across all neurons of one type. The maximal value for one firing probability hypothesis is therefore equal to the number of neurons of a given type, similar to *MSS*_*dir*_.

As an indicator for the total number of neurons with any direction sensitivity at all, we computed the expected absolute direction sensitivity score (ADSS):


(5) $$\begin{array}{l} \langle ADSS_{cat}\rangle =P\left(H\left(r_{m,A}>r_{s,A}\right)|D\right)*P\left(H\left(r_{m,B}<r_{s,B}\right)|D\right)\\ \;+P\left(H\left(r_{m,A}<r_{s,A}\right)|D\right)*P\left(H\left(r_{m,B}>r_{s,B}\right)|D\right) \end{array}$$

This score can take values between 0 and 1, with values close to zero indicating no direction selectivity and values close to one indicating direction selectivity, disregarding which motion direction elicits greater firing rates. We sum this score across all neurons of a given type.

The Supplementary material section ‘Statistical Model and Power Analysis of Motion Sensitivity' comprises a power analysis for the analyses of motion sensitivity and direction selectivity outlined above, indicating which difference in the recorded firing rates is considered evidence for the hypothesis that a neuron fires more in one of the two conditions.

### 2.5. Computational model

All computations were performed with the Python programming language (version 3.10.8) and the PyTorch (version 1.13.0) library. Plots were created with the Matplotlib library (version 3.6.2).

Our model comprises CL1a and CL2 neurons, adopting the projection schemes proposed by Heinze and Homberg (2008).

In contrast to a previous model of the CL1a-CL2 circuit (Pabst et al., 2022), the model described here also accounts for the reported arborization widths: No arborizations broader than one column were found in the PB. In the CBL, CL2 neurons innervate single columns. Ramifications of CL1a neurons, especially in the upper layers of the CBL, span up to five columns (Heinze and Homberg, 2008). These ramifications lead to an effective CL2 - CL1a connectivity in the CBL extending over up to five columns in the model. We assume that, as shown for E-PG and P-EN neurons in the fly (Turner-Evans et al., 2017), CL1a neurons provide synaptic inputs to CL2 neurons in the PB, which in turn provide synaptic inputs to CL1a neurons in the CBL. We further assume a combination of excitation and inhibition within the CL1a-CL2 connectivity instead of excitatory loops paired with global inhibition, as has been proposed for *Drosophila* (Turner-Evans et al., 2017). We refer to the model outlined thus far as the default model *Model*_*d*_ and introduce another version where all CL2 neurons from the same hemisphere are interconnected. This model is termed *Model*_*NO*_ as synapses giving rise to such a connectivity could occur in the lower units of the two NO (cf. **Figure 5**), which appears to be the case in *Drosophila* (Hulse et al., 2021). Since data on the excitatory and inhibitory nature of (proposed) synapses in the circuits modeled here are missing, all synaptic weights were determined via optimization with the objective of either maintaining or shifting compass activity. Initial weights are uniform for all excitatory and inhibitory connections, 0.5 and -0.5, respectively. They are set such that CL1a neurons excite CL2 neurons, which in turn inhibit CL1a neurons. Reversing this relation led to identical results after weight optimization. The firing rate neurons and synaptic connections in our model are linearized around their operating point, thus approximating their non-linear dynamics. We represent the CL1a-CL2 connectivity with matrices *M*_*d*_ and *M*_*NO*_ for the two versions of the model, *Model*_*d*_ and *Model*_*NO*_, respectively. For all neurons, the connectivity features additional self-recurrent connections and synapses onto neurons of the same type arborizing in adjacent PB columns to enable the maintenance of a baseline activity. The network's activity is characterized by deviations from a baseline firing rate, represented by a vector *x*_*t*_ with components *x*_*t*,1 : 16_ and *x*_*t*,17 : 32_ covering the CL1a and CL2 neurons, respectively. Vector components for each neuron type are ordered from left to right according to their PB column, which we label *L*8, …, *L*1, *R*1, …, *R*8. The network is recurrent and iterated across time steps such that the activity at the next time point, *t* + 1, can be computed from the activity at the current time point, *t*:


(6) $$\begin{array}{l} x_{t+1}=Mx_{t} \end{array}$$

#### 2.5.1. Maintenance of a stable head direction signal

In the framework outlined above, maintenance of the head direction representation or CL1a activity pattern *x*_1 : 16_ translates to an equality of *x*_*t*,1 : 16_ at time point *t* and *x*_*t*+1,1 : 16_ at the following time point, *t* + 1:


(7) $$\begin{array}{l} x_{t,1\;:\;16}=x_{t+1,1\;:\;16} \end{array}$$

According to Equation 6, this is given if *Mx*_*t*_ = *x*_*t*_. We refer to such *x*_*t*_ as stable states. We defined sinusoidal CL1a and CL2 activity targets ${\hat{x}}_{t,1:16}={\hat{x}}_{t,17:32}$ matching the tuning observed across the PB (Pegel et al., 2019; Zittrell et al., 2020). Each target had an activity maximum (“compass bump”) in one PB column. We used as many targets as there are PB columns in our model. For more details, see Pabst et al. (2022). We employed the L-BFGS algorithm (Liu and Nocedal, 1989) to optimize synaptic weights of *M*_*d*_ and *M*_*NO*_ by minimizing the mean-squared deviation between these targets and the network outputs over two time steps subject to the aforementioned arborization width constraints. Furthermore, we apply a weak quadratic synaptic weight regularization to push all non-essential connectivity to zero. Our results are robust against changes of the relative weight of the regularization, as long as it is ≈ 0.1 − 0.2.

#### 2.5.2. Rotation-induced shifts of the head direction signal

We tested two possible computational mechanisms that would produce a phasic shift from *x*_*t*_ to *x*_*t*+1_, representing the influence of rotational flow inputs on the compass system, putatively conveyed by TN or TB7 neurons: A purely feed-forward input exciting and/or inhibiting the CL1a and/or CL2 neurons and a modulatory input modifying the connectivity. We used the targets described above as initial network states. For both left and right turns, we defined targets ${\hat{x}}_{t+1,1:16}={\hat{x}}_{t+1,17:32}$ and ${\hat{x}}_{t+2,1:16}={\hat{x}}_{t+2,17:32}$ shifted in the direction opposing turn direction, such that the activity maximum or compass bump transitioned from one PB column to an adjacent one in each time step. For more details, see Pabst et al. (2022). Both feed-forward and modulatory inputs were optimized to minimize the mean-squared deviation between these shifted targets and the network outputs over two time steps using the L-BFGS algorithm subject to the aforementioned arborization width constraints and the weight regularization.

#### 2.5.3. Simulation

To test whether the learnt network parameters render a stable compass that can integrate an initial head direction signal with rotation inputs over a series of time points, we implemented an agent simulation. We simulated forward motion interrupted by a turn to the right followed by a turn to the left of equal magnitude. This trajectory was chosen to facilitate an intuitive understanding of the compass bump's traversal along the PB, including the 'wrapping around' at its lateral ends. Note that we only distinguish between movement directions at this point, assuming a uniform absolute angular velocity for all turns, which is not entirely biologically plausible. The starting compass bump position was set to an arbitrary PB column.

## 3. Results

We surveyed CX neurons at different integration stages for sensitivity to the moving gratings (Figures 1, 2). In total 62 morphologically identified neurons with arborizations in the CX were studied (Figure 1). These included 4 tangential input neurons (TL) to the CBL comprising the subtypes TL2 and TL3 (Figure 1A), 21 CL1a columnar neurons connecting the CBL to the PB, two CL2 columnar neurons connecting the PB, CBL and NO, five TB1 tangential neurons of the PB, three CPU1, seven CPU2 and one CPU5 neurons connecting distinct columns of the PB and CBU to the lateral complex (CPU1, CPU2) or a nodulus (CPU5), one CP1 and two CP2 neurons connecting the PB to distinct areas of the lateral complex (Figure 1B), eight PoU pontine neurons (Figure 1B), and various TU-type tangential neurons of the CBU (Figure 1A). We found sensitivity to the optic flow stimuli in some neural classes while others did not respond to the stimulation.

### 3.1. Sensitivity to translational and rotational optic flow in the central complex

**Figure 3:**
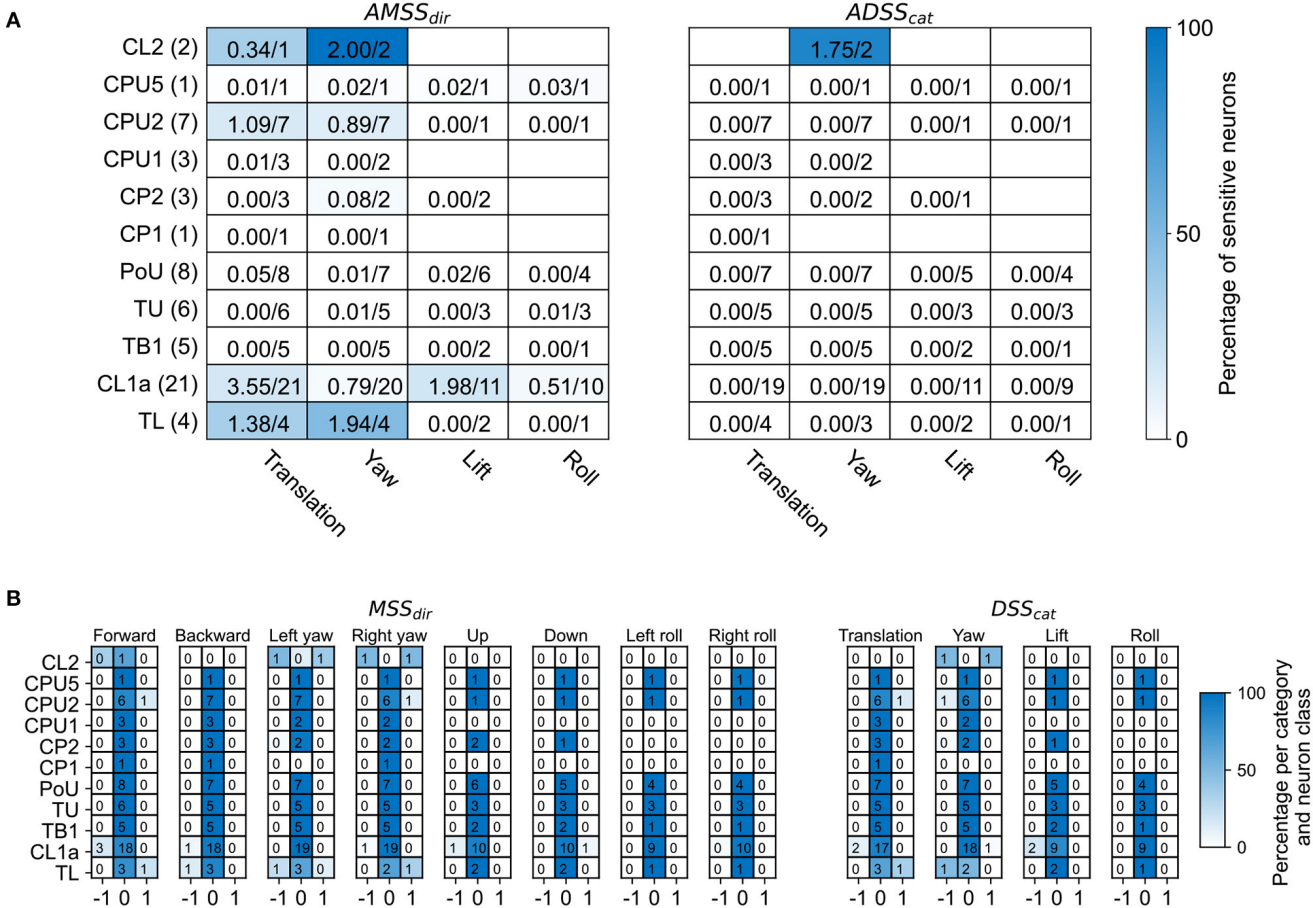
Overview of motion sensitivity and direction selectivity of all recorded neurons. **(A)** Absolute motion sensitivity scores per motion direction (*AMSS*_*dir*_, left) and absolute direction selectivity scores per motion direction category (*ADSS*_*cat*_, right), summed over neuron cell types. Absolute motion sensitivity scores take values between 0 and 1, with values close to 0 indicating no motion sensitivity and values close to 1 indicating motion selectivity, disregarding whether the neuron responds with an increase or decrease in activity. Absolute direction selectivity scores take values between 0 and 1, with values close to 0 indicating no direction selectivity and values close to 1 indicating direction selectivity, disregarding which motion direction elicits greater firing rates. Each cell holds the (rounded) sum of response scores over neuron cell types. Numbers are given as sums of scores over the total number of tested neurons. The fractions of summed scores and total possible scores are also indicated by the background color. The total number of recorded neurons for each neuron class is indicated in parentheses. Empty cells mean that no neuron was tested with the respective stimulus. **(B)** Distribution of motion sensitivity scores per motion direction (*MSS*_*dir*_, left) and direction selectivity scores per direction category (*DSS*_*cat*_, right), both per neuron class. Cell shading codes for the fraction of summed scores and total possible scores.

**Figure 4:**
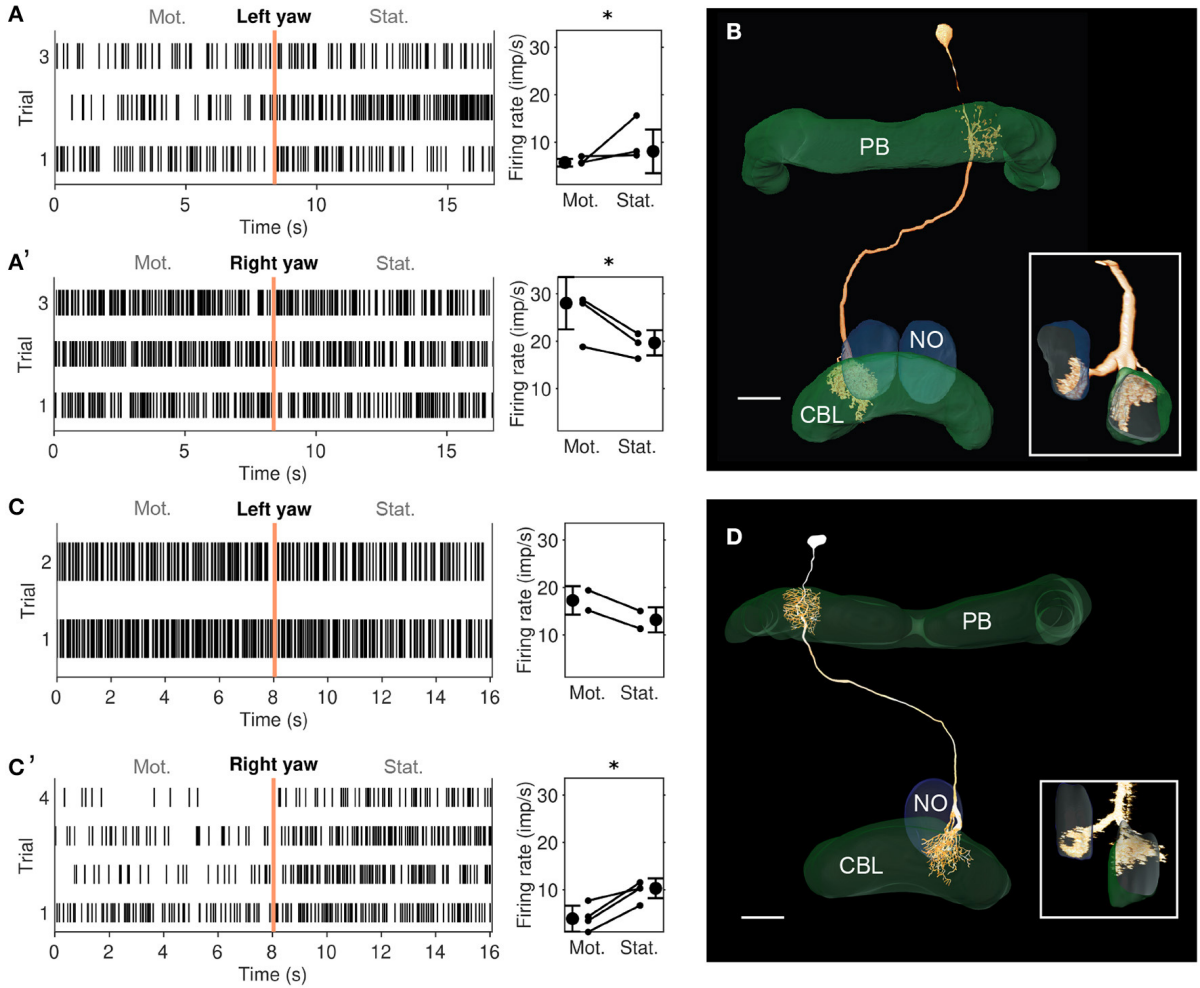
Physiological responses to yaw rotation and projections of CL2 neurons. **(A, A')** Physiological response (raster plots and mean firing rates) to left yaw rotation **(A)** and right yaw rotation **(A')** of the CL2 neuron shown in B (unit 801^*R*^ in Supplementary Figures S1, S2). The neuron shows reduced firing rate during simulated left yaw and increased firing activity during simulated right yaw. Vertical lines in the raster plots indicate onset of the stationary phase. An asterisk indicates “strong evidence” in favor of the hypothesis that the firing rates differ between the motion and stationary phases (i.e., it indicates a Bayes factor ≥10 according to the conventions established by Kass and Raftery 1995). **(B)** Skeleton view of the CL2 neuron (view from posterior) recorded in **(A, A')**. The neuron arborized in column R4 of the right hemisphere of the protocerebral bridge (PB), layers 1–3 of column L2 in the CBL, and in the lower unit of the left NO. Inset shows sagittal view of ramifications in the lower division of the central body (CBL), and the left nodulus (NO). Scale bar: 40 μm. **(C, C')** Raster plots and changes in firing rate during simulated yaw in the second CL2 neuron, shown in D (unit 800^*L*^ in Supplementary Figures S1, S2). The neuron increased its firing rate during simulated left yaw **(C)** and decreased its firing rate during simulated right yaw **(C')**. Like in A, an asterisk indicates 'strong evidence' for a firing rate difference between the motion and stationary phases. **(D)** Two-dimensional reconstruction of the neuron from confocal image stacks (view from posterior). It arborized in column L4 of the left hemisphere of the PB, column R2 in the CBL, and in the lower unit of the right NO. Inset shows sagittal voltex view illustrating ramifications in the CBL and NO. Scale bar: 40 μm.

Neurons in most of the examined morphological classes shown in Figures 1A–C were not sensitive to the moving gratings. Some of the tested TL-, CL1a-, and CPU2 neurons, however, were sensitive to grating patterns moving in at least one motion direction (motion sensitivity; Figures 3A, B). Response scores, indicating the sign of the firing rate change due to visual self-motion perception, were likewise inconsistently distributed within these neuron classes. Overall, within a given neuron class, individual neurons responded with excitation, inhibition or not at all to the same stimulus, independent of their brain side of origin (Figures 3A, B). Two CL2 neurons, however, were not only motion sensitive but also responded differently to opposing motion directions (direction selectivity, Figures 3A, B, 4, and Supplementary Figure S2).

### 3.2. Yaw-rotation is processed by CL2 neurons

We recorded from two mirror-symmetric CL2 neurons. One neuron had smooth, presumably postsynaptic arborizations in the left NO and in column R4 of the right half of the PB, and beaded processes in layers 1-3 of column L2 in the left half of the CBL (Figure 4B). The second CL2 neuron had ramifications in the right NO, column L4 in left half of the PB, and column R2 in the right half of the CBL (Figure 4D). Both neurons were directionally selective for visual motion that simulated yaw rotation, but with opposite polarity (Figures 4A, A', C, C' and Supplementary Figure S2). The CL2 neuron with arborizations in the right half of the PB and in the left NO (unit 801^*R*^, Supplementary Figures S1, S2) responded to right turns with an increase and to left turns with a decrease in firing rate, compared to baseline. The neuron was also weakly inhibited by forward motion. The CL2 neuron arborizing in the left half of the PB and the right NO (unit 800^*L*^ in Supplementary Figures S1, S2), on the other hand, responded to left turns with an increase and to right turns with a decrease in firing rate. Responses to translational motion stimuli were not tested. Neurons apparently homologous to CL2 in *Drosophila* (P-EN) signal rotational self-motion, updating the internal heading representation when the animal turns (Green et al., 2017; Turner-Evans et al., 2017).

**Figure 5:**
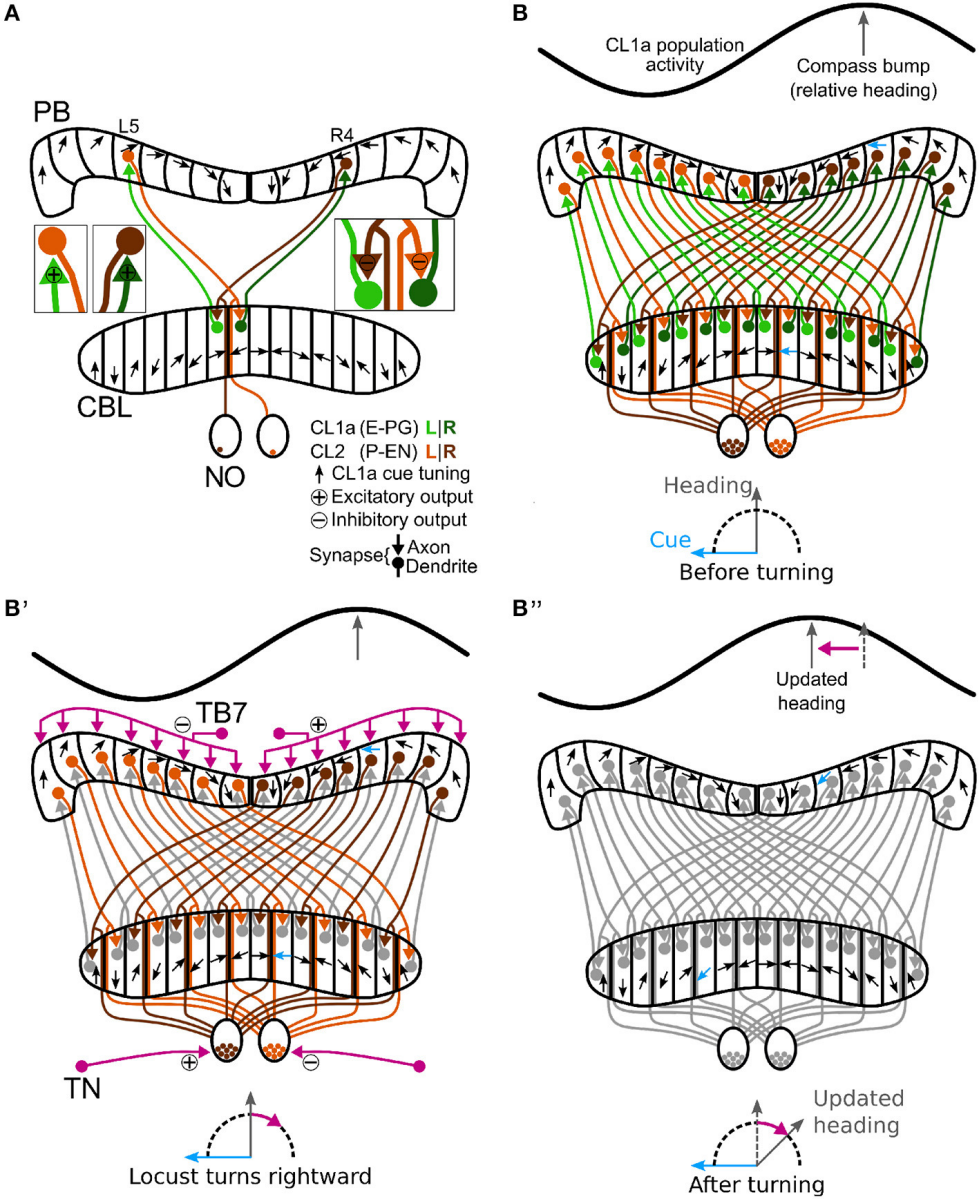
Schematic wiring diagram of CL1a and CL2 columnar neurons in the central complex and hypothetical shift mechanism of the internal heading signal in the PB. **(A)** Schematic wiring diagram of the CX with a subset of the involved neuron types: CL1a and CL2 neurons are connected to one another in the protocerebral bridge (PB) and lower division of the central body (CBL), while CL2 neurons also have postsynaptic arborizations in the noduli (NO). CL1a neurons are topographically tuned to solar azimuth along the PB (black arrows). **(B, B”)** Hypothetical shift mechanism of the internal heading signal in the PB. **(B)** Full population of CL1a and CL2 neurons and initial activity state in the network: With an environmental cue (sun) 90° left of the locust (bottom), the CL1a population activity (top) has a distinct maximum according to the neural tuning (highlighted arrows in PB and CBL). **(B')** When the locust turns right, CL2 neurons are excited or inhibited depending on their brain side. Neurons that innervate the left (right) NO are excited (inhibited) by tangential neurons (TN) from the lateral complexes and relay onto CL1a neurons from the left (right) half of the PB. This asymmetric input may analogously be conveyed in the PB by tangential neurons (TB7) from the superior posterior slope. **(B”)** After turning, the CL1a population activity maximum is shifted so that the neural heading estimate accordingly represents the new heading relative to the external cue. Wiring schemes from Heinze and Homberg (2008), topographic tuning in the PB and CBL based on Zittrell et al. (2020).

Although the physiological data on CL2 neurons are limited to only two recordings, which moreover could not be tested for responses to backward motion, lift and roll, the striking similarity in projection pattern between CL1/CL2 neurons in the locust and E-PG/P-EN neurons in the fly opens the possibility that the locust internal compass signal may, like in the fly, be shifted during turns via asymmetric excitation and inhibition of CL2 neurons (Figure 5B'). This idea is consistent with our simulation of compass shifts, as described below. The site of this interaction may either be the NO (via TN neurons) or the PB (via TB7 neurons). Both cell types are, like their equivalents in *Drosophila*, the GLNO neurons and the SpsP neurons (Hulse et al., 2021) morphologically suited to provide asymmetric input to the CL2 population. Like in *Drosophila* P-EN neurons, the projections of locust CL2 neurons in the CBL are shifted by one column relative to the projections of CL1 neurons (Figures 5A, B). A notable difference between compass representation in the locust and the *Drosophila* compass system is that the E-PG population activity peak in the EB results in two activity peaks with a fixed offset along the PB, while available data in the locust suggest a single peak along the PB that results from azimuthal tuning to celestial cues (Pegel et al., 2019; Zittrell et al., 2020). We refer to this single peak as the ‘compass bump'. If there is indeed a (single) compass bump, locust CL2 neurons might have inhibitory connections to CL1a neurons (Figure 5B). However, these connections and their polarity are hypothetical as there are no data on functional connectivity in the locust CX. Alternatively, the observed tuning could be a consequence of the projection and connectivity patterns of CL1a and CL2 neurons.

### 3.3. Computational model

#### 3.3.1. Maintenance of a stable head direction encoding

*Model*_*d*_ and *Model*_*NO*_ connectivities are based on the projection patterns described by Heinze and Homberg (2008), assuming synapses between CL1a and CL2 neurons arborizing at the same location. *Model*_*NO*_ further accounts for possible synapses within the two CL2 neuron subsets arborizing in the same nodulus, respectively. The proposed CL2-CL2 synapses are functionally equivalent to connections in *Drosophila* (Hulse et al., 2021). Both models can maintain an initial network activity pattern with the CL1a activity maximum or compass bump representing head direction relative to a global cue, such as the sun, when no yaw rotation is simulated.

**Figure 6:**
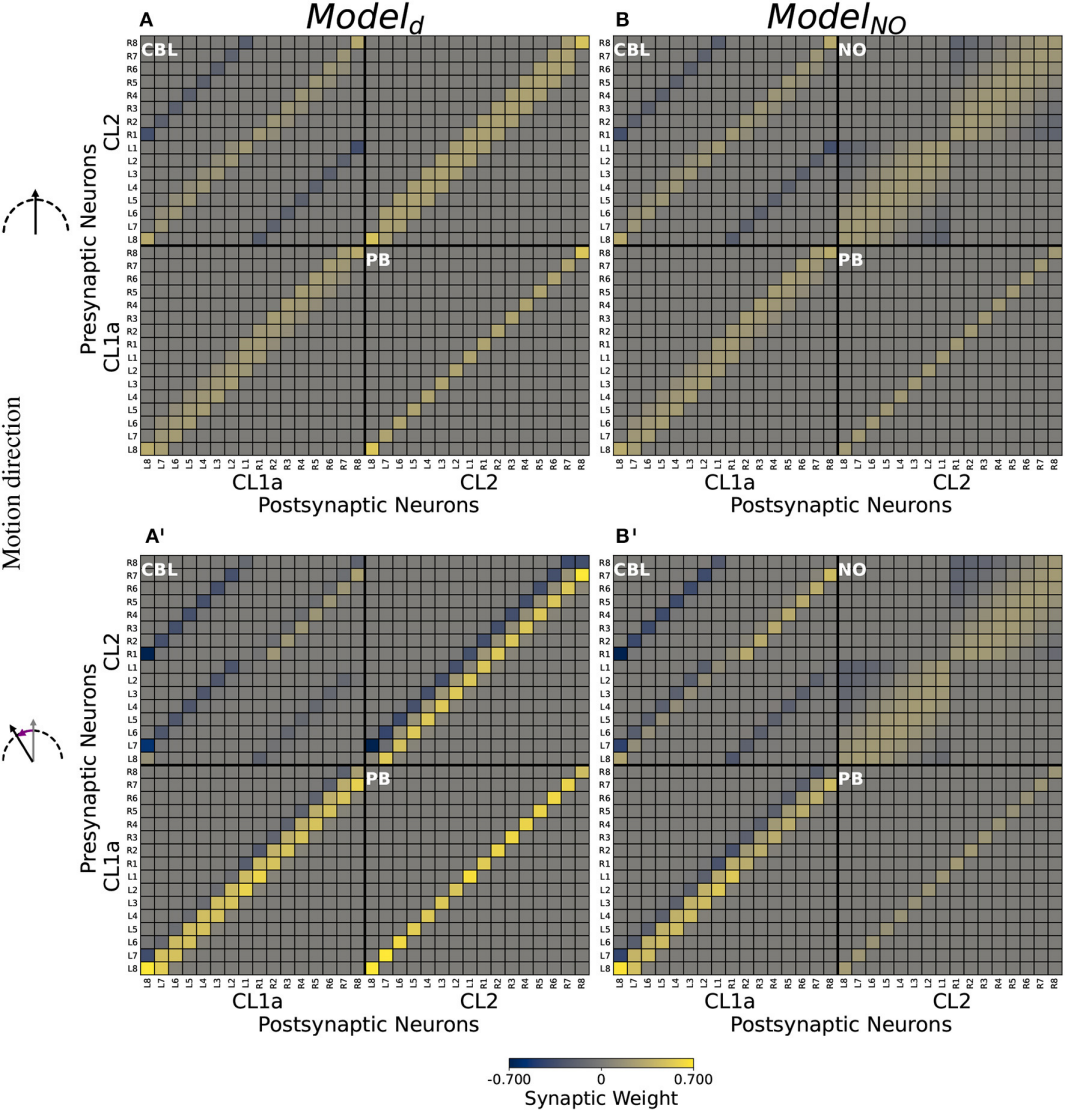
Computational model connectivities. **(A, B)** Connectivity matrices *M*_*d*_ and *M*_*NO*_ optimized for compass state maintenance. **(A', B')** Modulated connectivity matrices *M*_*d*_ and *M*_*NO*_ optimized for compass state shifts, depicted for left turns. For right turns, resulting modulated matrices are identical but with each quadrant rotated by 180°. Excitatory synapses are depicted in yellow, inhibitory synapses in blue. Neurons are indexed via the PB column (L8-R8) in which they arborize. Values are clipped at ±0.7 for better visibility.

For both model versions, optimization rendered all synapses from CL1a neurons onto CL2 neurons in the PB excitatory (cf. the lower right quadrants in Figures 6A, B respectively). In both model versions, CL2 neurons inhibit CL1a neurons projecting into the opposite hemisphere of the PB via connections in the CBL (cf. the two secondary diagonals in the upper left quadrants of Figures 6A, B, respectively) and excite CL1a neurons branching in the same PB hemisphere (cf. the main diagonals in the upper left quadrants of Figures 6A, B, respectively). CL1a-CL1a connectivities are similar in both models: In addition to the excitatory self-recurrent connection, CL1a neurons in adjacent PB columns are excited (cf. lower left quadrants in Figures 6A, B). In *Model*_*d*_, the CL2-CL2 connectivity resembles the CL1a-CL1a connectivity (cf. the upper right quadrant of Figure 6A). In *Model*_*NO*_, all CL2 neurons arborizing in the same PB hemisphere are potentially interconnected in the contralateral NO (cf. Figure 5B). Furthermore, inhibitory synapses exist in the noduli between CL2 neurons arborizing in opposite ends of each PB hemisphere (cf. the upper right quadrant of Figure 6B).

#### 3.3.2. Rotation-induced shifts of compass activity

Feed-forward input to the CL1a/CL2 neurons could not be optimized to induce compass bump shifts in *Model*_*d*_ or *Model*_*NO*_. However, the modulatory inputs were able to shift the bump (cf. Figures 6A', B' for modulated connectivities shifting the network activity to the right during left turns). The compass bump is shifted by modulations of the network connectivity at multiple sites: In both models, CL2 neurons asymmetrically excite or inhibit CL1a neurons branching in the same hemisphere of the PB (cf. the main diagonal in the upper left quadrants of Figures 6A', B'). In both models, CL1a neurons asymmetrically excite and inhibit neurons of the same type arborizing in adjacent PB columns. During left turns, neighbors to the left are excited and neighbors to the right are inhibited (cf. the lower left quadrants of Figures 6A', B'), and the opposite holds during right turns (not depicted). In *Model*_*d*_, the same applies to CL2 neurons (cf. the upper right quadrant of Figure 6A'). In *Model*_*NO*_ instead, a part of the inhibitory synapses among CL2 neurons is attenuated. During left turns, *CL*2_*L*8−*L*5_ and *CL*2_*R*1−*R*3_ less strongly inhibit *CL*2_*L*1−*L*3_ and *CL*2_*R*8−*R*6_, respectively (cf. the upper right quadrant of Figure 6B' compared to its counterpart in B). During right turns, this order is reversed: Inhibitory synapses from *CL*2_*L*1−*L*3_ and *CL*2_*R*8−*R*5_ onto *CL*2_*L*8−*L*5_ and *CL*2_*R*1−*R*3_, respectively, are attenuated (not depicted).

#### 3.3.3. Simulation

**Figure 7:**
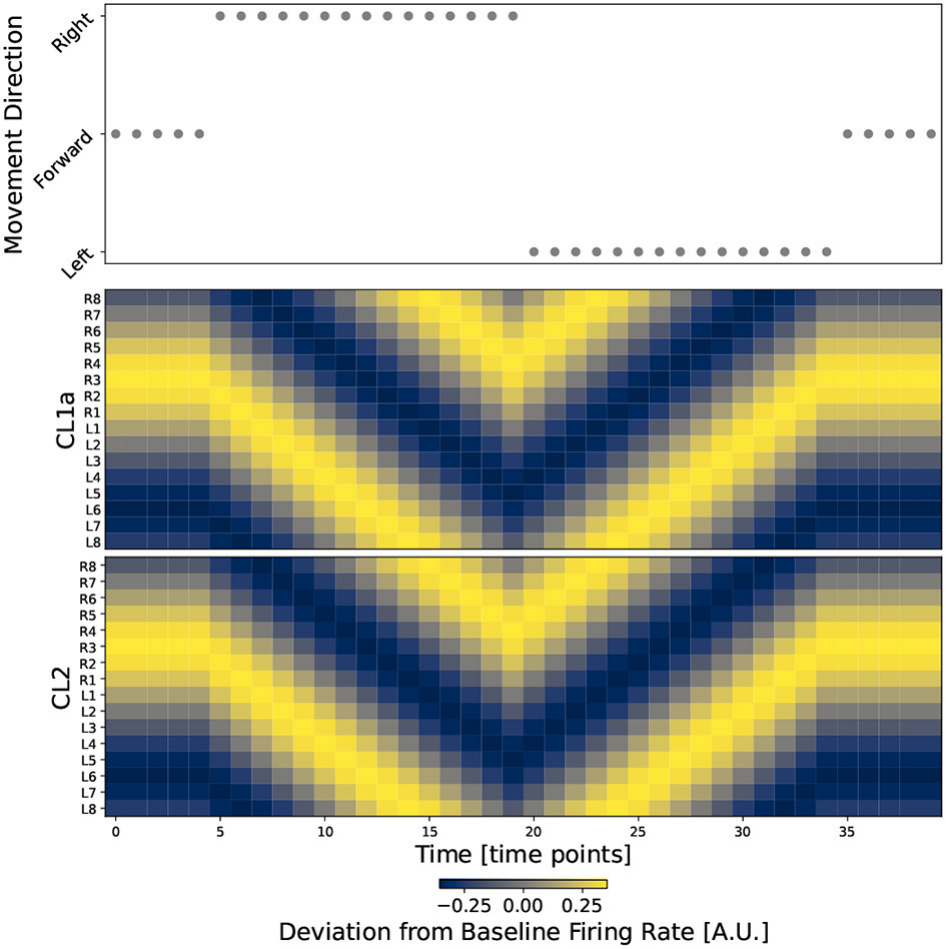
The circuit successfully integrates direction information into the heading signal. The top plot shows movement direction at discrete time points during a simulated walk. The two bottom plots show the firing rates of all CL1a and CL2 neurons in *Model*_*d*_, respectively. Neurons are indexed and arranged by their corresponding columns of the PB, revealing one activity bump along the PB in each subset of columnar neurons.

An example of *Model*_*d*_ in action is shown in Figure 7, where we simulate a heading trajectory and the resulting compass states. Results look identical for *Model*_*NO*_, see Supplementary Figure S3. The top panel shows the simulated motion directions and the two bottom panels depict the network activity at each time point. Activation of the CL1a and CL2 populations is equal at all time points, with one global activity maximum or bump along the PB in each subset of neurons. When the agent turns, both activity patterns are shifted in the direction opposing turning direction. The initial and final bump positions are identical, showing that direction information is integrated correctly across time. The compass bump can transition between the lateral ends of the PB: Between time points 13 and 14, the compass maximum moves from column L8 to R8, and a transition in the opposite direction happens between time points 23 and 24.

## 4. Discussion

We analyzed the sensitivity to visually simulated self-motion in different neuron classes of the locust CX network, from input-providing neurons (TL, TU neurons) to intermediate stage neurons (CL1a, CL2, POU, and TB1) and output neurons (CPU1, CPU2, CPU5, CP1, and CP2). Neurons in most of the investigated classes were not sensitive to visual self-motion. We hardly encountered consistent responses within the same neuron class, suggesting that single cells flexibly switch their cue sensitivity based on the internal state of the animal and environmental conditions (Shiozaki et al., 2020; Beetz et al., 2022; Fisher et al., 2022). Exceptions were CL2 neurons, which mirror-symmetrically encoded yaw rotation direction, depending on the brain hemisphere in which they arborized, suggesting a role in keeping the internal compass system up to date during turning.

A large fraction of cell types studied here (TL, CL1a, TB1, CPU1, CPU2, CP1, CP2) are elements of the sky compass system in the CX of the locust (Vitzthum et al., 2002; Heinze et al., 2009; Bockhorst and Homberg, 2015; Pegel et al., 2018; Zittrell et al., 2020). These neurons are sensitive to the azimuth of an unpolarized light spot (simulated sun) as well as to the polarization pattern above the animal (simulated sky) matching the position of the sun (Zittrell et al., 2020). The preference angles for solar azimuth in columnar neurons of the PB showed that solar azimuth is represented topographically across the columns of the PB as illustrated in Figure 5. The lack of responses to large-field motion stimuli in most of these neurons is in contrast to data from Rosner et al. (2019), who showed that a majority of sky compass neurons in the locust CX (types TL, CL1, TB1, CPU1, CPU2) were sensitive to progressive motion simulated through moving gratings. The reason for these different results most likely lies in different preparations of the animals. While in this study, legs and wings were removed, animals in the study of Rosner et al. (2019) had their legs attached and could perform walking motion on a slippery surface. Therefore, while the responses to sky compass signals may be affected only mildly, differences in behavioral context and internal state apparently play a major role for the sensitivity of sky compass neurons to visually simulated self-motion. Neurons of the CBU (PoU, TU, CPU5) that are not directly involved in sky compass signaling, were, likewise, unresponsive to visual self-motion. This coincides with studies on *Drosophila* that found that responsiveness of neurons of the fan-shaped body (corresponding to the locust CBU) to motion stimuli highly depended on whether the animals were actively engaged in flight (Weir and Dickinson, 2015; Shiozaki et al., 2020). It is therefore likely, as for neurons of the sky compass system, that neurons at this integration stage are silent in locusts under the constrained conditions of our experiments. HΔb neurons in *Drosophila* (corresponding to PoU neurons in the locust) integrate external and internal self-motion cues to transform egocentric directions into world-centric coordinates (Lu et al., 2022; Lyu et al., 2022). The lack of mechanosensory feedback under our experimental conditions likely explains why PoU neurons did not respond to purely visual self-motion cues. Under such conditions, PoU neurons and others, instead, strongly respond to looming objects (Rosner and Homberg, 2013), thus they might rather be involved in escape reactions when quiescence is signaled by the body. In general, physiological activity of locust CX neurons is considerably affected by active leg movement (Rosner et al., 2019). In our study, the legs were cut off, eliminating any proprioceptive sensory feedback.

In contrast to the lack of responsiveness in most cell types, two mirror-symmetric CL2 neurons showed robust responses to simulated yaw rotation with opposite directional preference. Inspired by the proposed role of P-EN neurons in *Drosophila* (corresponding to CL2 neurons in the locust) in updating and shifting the activity peak across the columns of the PB, we developed a computational model testing the likely function of CL2 neurons in the locust. The model of the CL1a-CL2 network resembles the recurrent loop connectivity between E-PG and P-EN neurons accounting for angular velocity integration in the *Drosophila* CX (Turner-Evans et al., 2017, 2020; Hulse et al., 2021). However, distinct differences exist, based on the 360° angular representation in the locust PB (Pegel et al., 2019; Zittrell et al., 2020) compared to the 2 × 360° representation of space in the *Drosophila* PB. While in *Drosophila* E-PG neurons form a 360° representation of space in the ellipsoid body, two opposite 180° representations of space would be topographically intercalated in the CBL of the locust (Figure 5A). In *Drosophila* P-EN and E-PG neurons are connected by recurrent excitatory loops with additional global inhibition (Turner-Evans et al., 2017, 2020). In the locust, instead, both inhibitory and excitatory connections between CL1a and CL2 neurons are required for compass state maintenance, see Figure 6.

Physiological data revealing the relationship between the activities of these two populations would aid model evaluation and refinement. Close to equal E-PG and P-EN bump positions have been found in *Drosophila* moving at a low angular velocity, with an offset increasing with angular velocity (Turner-Evans et al., 2017). Neither of our model versions could perform a shift of compass activity with a feed-forward input only, which might be due to the fact that our models do not include a closed loop from one end of the PB/CBL to the other. The inclusion of further neuron types might in fact close this gap and is the prospect of future work. CL1b-d neurons (Heinze and Homberg, 2008; Heinze et al., 2009) might, in addition, further stabilize the compass representation during standstill or forward motion. An internal compass representation must adapt to a new heading direction when the animal turns. In the CX, this is likely accomplished by integrating rotation cues of different modalities. Two entry sites into the CX network for information on rotational self-motion have been proposed so far, based on work in the fruit fly: i) The PB, where neurons may receive asymmetric input excited depending on turning direction, conveyed via IbSpsP neurons (TB7 neurons in the locust) (Hulse et al., 2021). These neurons connect specifically to P-EN neurons (CL2 neurons in the locust). ii) The NO, where GLNO neurons (TN neurons in the locust) that receive input in the lateral complex and innervating one NO might be excited/inhibited depending on turning direction. P-EN neurons convey these asymmetric inputs to E-PG neurons via synapses in the ellipsoid body, leading to a shift of the internal heading representation according to turning (Green et al., 2017; Turner-Evans et al., 2017). We explored two possible network connectivities and two possible mechanisms inducing the compass bump shift on an algorithmic level.

Based on the projection patterns of CL1a and CL2 neurons described by Heinze and Homberg (2008), we assumed that an axon and dendrite are synaptically connected if they arborize at the same location. In the default model *Model*_*d*_, we did not assume CL2-CL2 connections within the two NO, but *Model*_*NO*_ allowed for such connections. They could occur in the lower units of the two NO, in *Drosophila* functionally equivalent connections appear to be present (Hulse et al., 2021).

Synaptic weights were initialized such that CL1a neurons excite CL2 neurons which in turn inhibit CL1a neurons, and excitatory self-recurrent connections were added among both subpopulations. As data supporting these assumptions are missing, all synaptic weights were optimized such that the models would maintain a stable network activity in the absence of any inputs. Both models could be optimized to maintain stable compass states.

Modulations of the network connectivity could be optimized to bring about compass bump shifts in both model versions: Shifts of the network activity are mediated by CL1a and CL2 neurons asymmetrically exciting and inhibiting neurons of the same type arborizing in adjacent PB columns in a direction-dependent manner. In *Model*_*NO*_, shifts are additionally mediated by asymmetrically attenuating inhibitory synapses between CL2 neurons arborizing at opposite ends of the same PB hemisphere. Note that the connectivity among neurons of the same type implemented here is most likely an abstraction of the effective connectivity which is likely mediated by neurons of other types not included in this model. Simulating an abstracted heading trajectory, we demonstrated that both model versions can integrate motion direction-dependent inputs to update a heading signal encoded in the network activity pattern. The networks can shift the compass bump from one lateral end of the PB to the other, indicating compatibility with a ring-attractor functionality also described in other species. Connectomics data would be necessary to evaluate which model version to prefer over the other.

In our models, the bump is not shifted by lateral transport of neuronal activation. Rather, during turns the connections of CL1a/CL2 neurons to CL1a/CL2 neurons in neighboring columns are up- or down-regulated depending on the turn direction. This leads to a corresponding change of the neuronal activation that yields a compass bump shift. For example, during a left turn of the animal, the compass bump is shifted right (see Figure 7, from 20-30 seconds). A right shift of the bump means that activities on the rising slope of the bump, viewed from left to right, must decrease. Conversely, activities on the falling slope must increase. This effect is brought about by computing the difference between activations in the neighboring columns, i.e. a given CL1a neuron needs to receive inhibitory input from its right neighbor, and excitatory input from its left neighbor (see Figures 6A', B'). During right turns, the modulation is reversed. While this mechanism does not require a ring closure in the network, such a closure would be necessary for a lateral transport of neuronal activation. As mentioned above, it is conceivable that the consideration of further neuron types in the future will render the compass network of the desert locust closed, and modeling could be employed to explore possible mechanisms of activation transmission among the involved neuron populations. Franconville et al. (2018) reported that connections from E-PG onto P-EN neurons in the PB are mediated by Δ7 neurons. As TB1 and TB2 neurons cross the midline of the locust PB, they are, in addition to contralateral processes observed in some CL1 neurons innervating the innermost columns of the PB (Sayre et al., 2021), candidates for mediating ring closure.

The linear model and discrete motion steps employed here are still quite abstract representations of the neuronal and behavioral characteristics of the locust. So far, our model is not dynamic; it switches between stable states but does not make the dynamics underlying the transitions explicit. We aim to increase the model's biological plausibility by implementing velocity dependence in future work but expect the general principles of maintaining and updating the compass bump to hold independently of the level of analysis.

## Data availability statement

The datasets analyzed and generated for this study along with the code written for analysis and modeling can be found in the data_UMR repository (http://dx.doi.org/10.17192/fdr/76).

## Author contributions

FZ, RR, and UH designed the experiments. FZ, EC, UP, and RR performed the experiments. FZ wrote manuscript. KP and DE designed the computational model and statistical analysis. KP revised the manuscript, analyzed the data, and implemented the computational model with DE. DE and UH conceived, designed, and directed research and helped write the manuscript. All authors contributed to the article and approved the submitted version.
