# Supplementary material for: Integration of optic flow into the sky compass network in the brain of the desert locust
Source: Front Neural Circuits. 2023 Apr 28;17:1111310. doi: 10.3389/fncir.2023.1111310 (PMC10175609; doi:10.3389/fncir.2023.1111310)
Supplement: Supplementary file 1 [file Data_Sheet_1.pdf]

# Supplementary Material

## 1 SUPPLEMENTARY TABLES AND FIGURES

### 1.1 Individual Motion Sensitivity Scores

We have described the motion sensitivity of a neuron by the posterior distribution over the hypotheses $H \in \{H(r_m < r_s), H(r_m == r_s), H(r_m > r_s)\}$ that the firing probability $r_m$ during motion stimulation is less/equal/more than the firing probability $r_s$ during stationary stimulation, see section 'Motion Sensitivity' of main paper. To summarize the information embedded in this posterior distribution per neuron, we computed the posterior expectation of the motion sensitivity score (MSS) per neuron and motion direction (dir):

$$\langle MSS_{dir} \rangle = P(H(r_m > r_s)|D) - P(H(r_m < r_s)|D) \quad \text{(S1)}$$

This score can take values between -1 and +1, with negative values indicating decreased firing and positive values indicating increased firing during the motion phase compared to the stationary phase. Furthermore, because this score represents a probability difference, $P(H(r_m > r_s)|D) \geq MSS_{dir}$. This implies that we can be at least as sure as $\langle MSS_{dir} \rangle$ of an increased motion response relative to the baseline, and conversely for negative scores. Values close to zero can result from equal activity during both phases (a high posterior for hypothesis $H(r_m == r_s)$) as well as from uncertainty about the three hypotheses (similar probabilities assigned to hypotheses $H(r_m < r_s)$ and $H(r_m > r_s)$ ). Therefore, we also computed an expected absolute motion sensitivity score (AMSS) for each direction:

$$\langle AMSS_{dir} \rangle = 1 - P(H(r_m == r_s)|D) \quad \text{(S2)}$$

This score can take values between 0 and +1, with values close to zero indicating no motion sensitivity and values close to one indicating motion sensitivity, irrespective of whether the motion response is a decrease or increase in firing compared to the stationary phase.

Supplementary Figure S1 shows the $\langle MSS_{dir} \rangle$ and the $\langle AMSS_{dir} \rangle$ for all neurons which we evaluated. The largest proportion of motion-sensitive neurons was found among CL1a, CL2, and TL neurons.

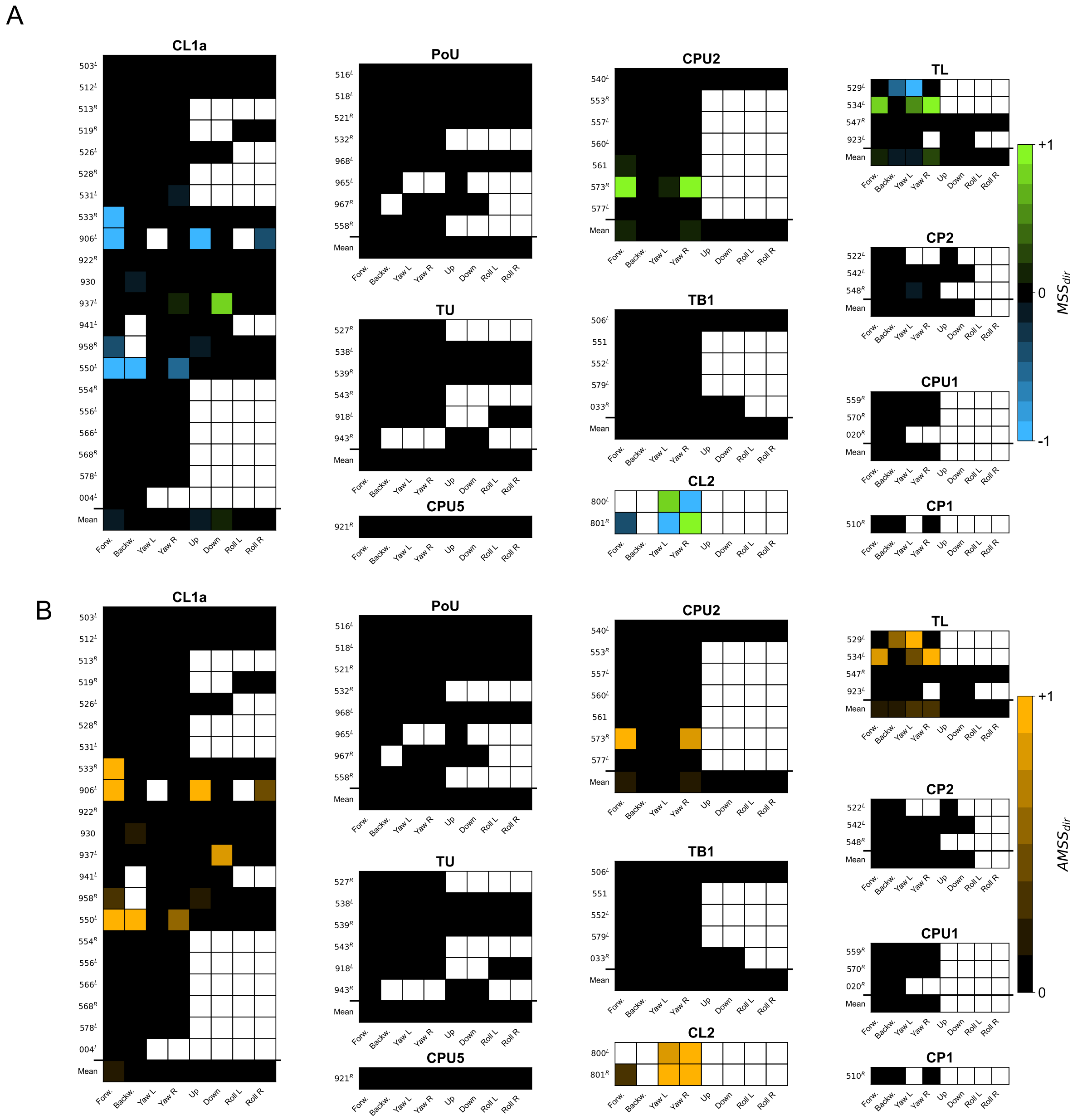


**Supplementary Figure S1.** Individual motion sensitivity scores per motion direction and neuron class. **(A)** False-color matrices show individual response scores for expected motion sensitivity $\langle MSS_{dir} \rangle$, indicating whether neurons responded to a given motion direction. Individual response scores range from -1 (strong inhibition compared to stationary stimulation, solid blue) to +1 (strong excitation, solid green) with 0 indicating a response that was best explained by $H(r_m == r_s)$, i.e. unchanged by motion (solid black). The 'mean' row, which was computed for more than four neurons of a type, holds the average response score per column, indicating whether neurons in the respective group tended to respond consistently. Rows are sorted for average response score; row numbers denote neuron ID, superscripts denote the brain side of the soma position. Empty fields indicate that the neuron was not tested with the corresponding stimulus; these fields did not contribute to the column average. **(B)** Same as A but for expected absolute motion sensitivity score $\langle AMSS_{dir} \rangle$, which ranges from +1 (strong firing rate change, solid orange) to 0 (no firing rate change, solid black). 'Mean' row has the same meaning as in **A**. Figure 1 of the main paper shows raw data of neuron $550^L$ (CL1a).

## 1.2 Individual Direction Selectivity Scores

For a per-neuron summary of direction selectivity, we computed the posterior expectation of the direction sensitivity scores (DSS) for each motion category cat $\in \{\text{translation}, \text{yaw}, \text{lift}, \text{roll}\}$ (see section 'Direction Selectivity' of main paper):

$$\begin{aligned} \langle DSS_{cat} \rangle &= P(H(r_{m,A} > r_{s,A})|D) * P(H(r_{m,B} < r_{s,B})|D) \\ &- P(H(r_{m,A} < r_{s,A})|D) * P(H(r_{m,B} > r_{s,B})|D) \end{aligned} \quad \text{(S3)}$$

Here, $A, B$ are the opposite motion directions in each motion category:

| category | A | B |
|---|---|---|
| translation | forward | backward |
| yaw | left | right |
| lift | up | down |
| roll | left | right |

$\langle DSS_{cat} \rangle$ can take values between -1 and +1, with negative values indicating decreased firing and positive values indicating increased firing during the motion A phase compared to the motion B phase. Values close to zero can result from equal responses to both directions as well as from uncertainty about (meaning similar probabilities assigned to) hypotheses $H(r_m < r_s)$ and $H(r_m > r_s)$ indicating opposing responses. We therefore also computed the expected absolute direction sensitivity score (ADSS) per neuron, as an indicator for any firing rate changes between motions in opposing directions:

$$\begin{aligned} \langle ADSS_{cat} \rangle &= P(H(r_{m,A} > r_{s,A})|D) * P(H(r_{m,B} < r_{s,B})|D) \\ &+ P(H(r_{m,A} < r_{s,A})|D) * P(H(r_{m,B} > r_{s,B})|D) \end{aligned} \quad \text{(S4)}$$

Supplementary Figure S2 shows the $\langle DSS_{cat} \rangle$ and the $\langle ADSS_{cat} \rangle$ for all neurons which we evaluated. Only CL2 neurons exhibited direction selectivity.

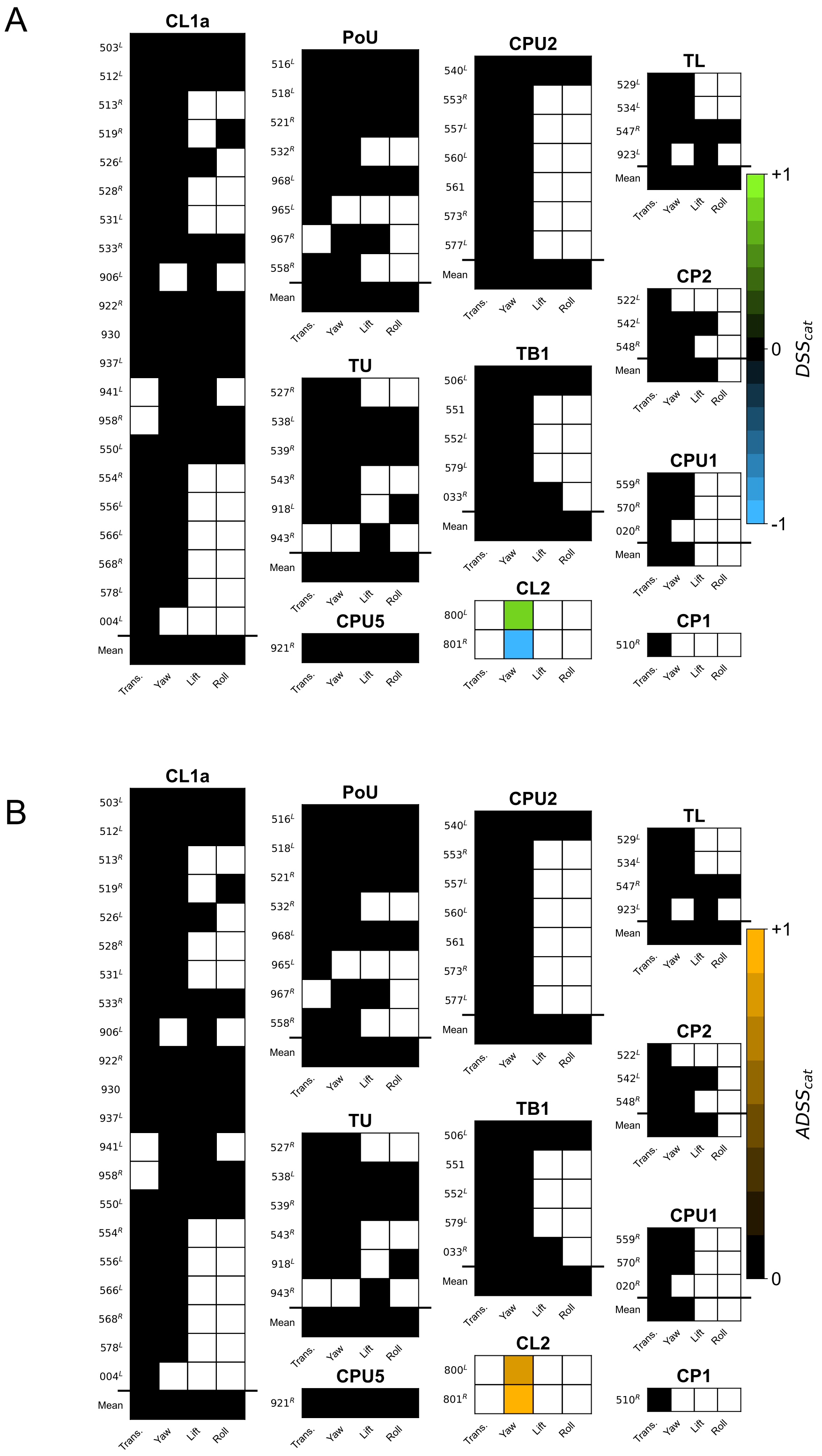


**Supplementary Figure S2.** Individual direction selectivity scores per direction category and neuron class. **(A,B)** Analogous to S1 but showing posterior expectations of direction selectivity scores $DSS_{cat}$ and $ADSS_{cat}$. The former indicates whether neurons responded by increased or decreased firing rate changes to opposing motion directions. The latter indicates whether the neuron responded to opposing motion directions at all.

### 1.3 Agent simulation with $Model_{NO}$

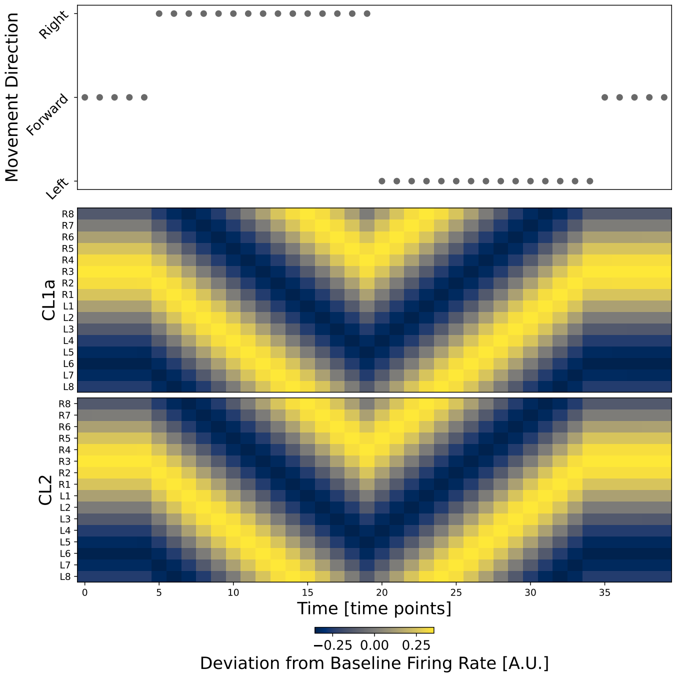


**Supplementary Figure S3.** The circuit successfully integrates direction information into the heading signal. The top plot shows movement direction at discrete time points during a simulated walk. The two bottom plots show the firing rates of all CL1a and CL2 neurons in $Model_{NO}$, respectively. Neurons are indexed and arranged by their corresponding columns of the PB, revealing one activity bump along the PB in each subset of columnar neurons. The relative weight of the synaptic weight regularization was 0.2 in this simulation.

## 2 STATISTICAL MODEL AND POWER ANALYSIS OF MOTION SENSITIVITY

We designed a Bayesian model for the evaluation of the experimental spiking data, to test the hypotheses that the firing probability of a motion phase $r_m$ is smaller, equal or larger than the firing probability $r_s$ during a stationary phase. We denote these hypotheses by $H \in \{H(r_m < r_s), H(r_m == r_s), H(r_m > r_s)\}$. Given the firing probabilities, we assume that the data $D = (s_m, g_m, s_s, g_s)$ of one experiment, comprised of spikes $s_m, s_s$ during motion/stationary phases and corresponding non-spikes/gaps $g_m, g_s$, are generated by a Bernoulli process with a refractory period of 2 ms, which is typical for the neurons we investigate. There might be additional dependencies between spikes that are not captured by a refractory Bernoulli process, but these are not relevant for our hypotheses. The Bernoulli observation probability is given by

$$P(D|r_m, r_s) = r_m^{s_m}(1 - r_m)^{g_m} \cdot r_s^{s_s}(1 - r_s)^{g_s} \tag{S5}$$

Since we are interested in hypotheses about firing probabilities relationships, we define a joint symmetric Beta prior with parameters $\alpha, \beta$ on $r_m$ and $r_s$, constrained by the hypothesis we wish to evaluate. We choose a symmetric prior to avoid a-priori biases beyond $H$. For $H(r_m < r_s)$, this prior is

$$P(r_m, r_s|\alpha, \beta, H(r_m < r_s)) \propto B(r_m|\alpha, \beta)B(r_s|\alpha, \beta)\mathbb{I}(r_m < r_s) \tag{S6}$$

where $B(r_m|\alpha,\beta)$ is a Beta density in $r_m$ and $\mathbb{I}(r_m < r_s)$ is an indicator function which is 1 if the condition in the parentheses is true, and 0 otherwise. This indicator function ensures that only hypothesis-conforming $r_m, r_s$ pairs have nonzero probability. The constant of proportionality can be obtained from the requirement that the prior be normalized. Thus, this prior can be written as

$$P(r_m, r_s|\alpha, \beta, H(r_m < r_s)) = \frac{2}{B(\alpha,\beta)^2} r_m^{\alpha-1}(1-r_m)^{\beta-1} r_s^{\alpha-1}(1-r_s)^{\beta-1}\mathbb{I}(r_m < r_s) \tag{S7}$$

The prior resulting from $H(r_m > r_s)$ can be obtained by inversion of the $<$ in the indicator function, whereas the prior for $H(r_m == r_s)$ is simply one Beta prior for both (equal) firing probabilities.

Since we are largely ignorant about the values of $\alpha$ and $\beta$, we chose these parameters by maximizing the differential entropy subject to the condition that the average firing probability is $\approx 0.05$ in a 2 ms time bin, which is typical for our neurons. We found $\alpha = 0.96$ and $\beta = 18.28$, and used these values for the rest of the analysis.

To compute the hypothesis posterior

$$P(H|D) = \frac{P(D|H)P(H)}{\sum_H P(D|H)P(H)} \tag{S8}$$

via Bayes' rule, we chose a uniform hypothesis prior $P(H) = \frac{1}{3}$. We evaluated the probability $P(D|H)$ by marginalizing the firing probabilities using Equation S5 and Equation S7. For example, letting $H = H(r_m < r_s)$:

$$\begin{aligned}
P(D|H(r_m < r_s)) &= \int_0^1 dr_s \int_0^1 dr_m P(D, r_m, r_s|H(r_m < r_s)) \\
&= \int_0^1 dr_s \int_0^1 dr_m P(D|r_m, r_s) P(r_m, r_s|H(r_m < r_s)) \\
&= \frac{2}{B(\alpha,\beta)^2} \int_0^1 dr_s \int_0^1 dr_m r_m^{\alpha+s_m-1}(1-r_m)^{\beta+g_m-1} r_s^{\alpha+s_s-1}(1-r_s)^{\beta+g_s-1}\mathbb{I}(r_m < r_s) \\
&= \frac{2}{B(\alpha,\beta)^2} \int_0^1 dr_s \int_0^{r_s} dr_m r_m^{\alpha+s_m-1}(1-r_m)^{\beta+g_m-1} r_s^{\alpha+s_s-1}(1-r_s)^{\beta+g_s-1} \\
&= \frac{2B(\alpha+s_m, \beta+g_m)}{B(\alpha,\beta)^2} \int_0^1 dr_s r_s^{\alpha+s_s-1}(1-r_s)^{\beta+g_s-1} I_B(r_s, \alpha+s_m, \beta+g_m)
\end{aligned} \tag{S9}$$

where $I_B(r, \alpha, \beta)$ is an incomplete beta function in $r$ with parameters $\alpha, \beta$. We solved the last integral by Taylor-expanding $I_B(r_s, \alpha+s_m, \beta+g_m)$ to second order at $\frac{\alpha+s_s}{\alpha+s_s+\beta+g_s}$, which yields a good approximation as long as $s_m \approx s_s$ and $g_m \approx g_s$. This is the case in our data.

The probability $P(D|H(r_m > r_s))$ can be evaluated by simply switching the roles of $r_m$ and $r_s$ in the above derivation. For $P(D|H(r_m == r_s))$, where there is only one rate, the integrals can be solved analytically to yield the well-known result

$$P(D|H(r_m == r_s)) = \frac{B(\alpha + s_m + s_s, \beta + g_m + g_s)}{B(\alpha, \beta)}. \tag{S10}$$

To facilitate interpretation of the values of the absolute motion sensitivity (AMSS, see section 'Statistical Analysis' of the paper) and the motion hypothesis posterior, which we use to average the motion sensitivity scores (MSS), we conducted a power analysis. We simulated 10,000 repetitions of a typical experiment in our study, where an animal is stimulated for 5 s with either stationary or motion input. We generated spikes according to the Bernoulli process assumption with 2 ms time bins by drawing spike counts from a binomial distribution. The firing rate of the stationary phase was set to 25 Hz, which corresponds to a firing probability $r_s = 0.05$ and $N = 2500$ Bernoulli trials during a single run of the experiment. An experiment consisted of five simulated runs in the simulation. The firing probability during the motion phase was assumed to be a multiple of $r_s$ in the range $1.15 \ldots 1.30$. This range is covered by a strongly responding neuron, see e.g. Figure 4C' of the main paper, right panel. To relate our motion sensitivity scores to standard measures used in statistical contexts, we evaluated the Bayes factor in favor of a changed firing rate during motion:

$$BF(r_m \neq r_s) = \frac{P(D|H(r_m > r_s)) + P(D|H(r_m < r_s))}{P(D|H(r_m == r_s))} \tag{S11}$$

The simulation results are shown in Figure S4. The top panel shows the AMSS, the middle panel the corresponding Bayes factors. The dotted lines show the boundaries for weak and strong evidence according to Kass and Raftery (1995). For strong evidence, the firing rate ratio has to be greater than 1.25, which implies an average AMSS $> 0.65$. In the bottom panel, we plotted the hypothesis posterior, which we use for averaging of the MSS. Strong evidence for an increased firing rate (MSS=+1 in Figure 3B of the paper) requires MSS $> 0.65$.

## REFERENCES

Kass, R. E. and Raftery, A. E. (1995). Bayes factors. *Journal of the American Statistical Association* 90, 773–795. `https://doi.org/10.2307/2291091`

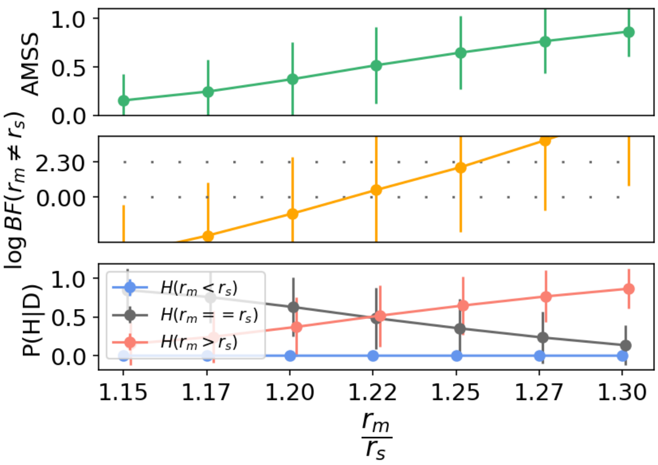


**Supplementary Figure S4.** Power analysis of the Bayesian hypothesis comparison used for motion sensitivity analysis. The circles and error bars are means and standard deviations computed across 10,000 repetitions of a simulated experiment. The ratio of the motion phase firing rate $r_m$ and $r_s$ is shown along the abscissa. **Top**: absolute motion sensitivity (AMSS, see paper for definition). **Middle**: Bayes factor in favor of the hypothesis that the firing probabilities/rates are different during motion vs. equal rates, larger values represent stronger evidence. The dotted lines show the boundaries for weak and strong evidence according to Kass and Raftery (1995). **Bottom**: hypothesis posterior, used for the averaging of the motion sensitivity score (MSS). The certainty of $H(r_m > r_s)$ increases with an increasing $\frac{r_m}{r_s}$ ratio. $\frac{r_m}{r_s} \approx 1.25$ is sufficient for strong evidence on average. For details, see text.
